# Refractory CrMoNbWV High-Entropy Alloy Manufactured by Mechanical Alloying and Spark Plasma Sintering: Evolution of Microstructure and Properties

**DOI:** 10.3390/ma14030621

**Published:** 2021-01-29

**Authors:** Nikolay Razumov, Tagir Makhmutov, Artem Kim, Boris Shemyakinsky, Aleksey Shakhmatov, Vera Popovich, Anatoly Popovich

**Affiliations:** 1 Institute of Machinery, Materials, and Transport, Peter the Great St. Petersburg Polytechnic University, 195251 St. Petersburg, Russia; n.razumov@onti.spbstu.ru (N.R.); artem_7.kim@mail.ru (A.K.); borisshe@gmail.com (B.S.); alexey.shax@gmail.com (A.S.); V.Popovich@tudelft.nl (V.P.); director@immet.spbstu.ru (A.P.); 2 Department of Materials Science and Engineering, Delft University of Technology, 2628 Delft, The Netherlands

**Keywords:** high entropy alloys, spark plasma sintering, mechanical alloying, wear, corrosion behavior

## Abstract

In this study, bulk samples of a CrMoNbWV high-entropy alloy (HEA) were obtained for the first time by spark plasma sintering (SPS) of mechanically alloyed (MA) powders at 1200 °C, 1300 °C, and 1400 °C. Microstructure evolution, phase formation as well as wear and corrosion behavior were investigated. The MA powders’ phase composition was found to be represented by body-centered-cubic (BCC) solid solution. The solid solution partially decomposed to Laves phases under the sintering, such as Cr_2_Nb and (Fe, Cr)Nb, and NbVO_4_-VO oxides mixture. The temperature increase to 1400 °C led to a grain coarsening of the BCC phase and decreased the Laves phase content accompanied by precipitation at the grain boundaries. The sintered samples showed high hardness and compressive strength (2700–2800 MPa) at room temperature. The wear tests demonstrated excellent results in comparison to conventional wear-resistant composites. The obtained samples also exhibited high corrosion resistance under electrochemical tests in H_2_SO_4_ solution. The CrMoNbWV HEA has comparable mechanical and corrosive properties with the WNbMoTaV type HEA, but at the same time has a reduced density: CrMoNbWV—10.55 g/cm^3^, WNbMoTaV—12.42 g/cm^3^.

## 1. Introduction

One of the most promising and progressive fields in modern material science is high-entropy alloys (HEAs). The first to propose HEAs’ concept was J.W. Yeh [1], but since then, it has changed rather significantly, especially during the last few years [2]. Due to their extremely high mechanical and physical properties, refractory HEAs are classified as a separate group of materials. Such alloying compositions mainly include elements from the IV, V, and VI columns of the periodic table with some additions of Al, Si, Ni, or Co [3].

First high-entropy equiatomic compositions based on refractory elements, such as NbMoTaW and NbMoTaWV, were obtained in prior research [4,5]. It was found that the alloys possess higher mechanical properties at higher temperatures in comparison to conventional superalloys, such as Inconel 718 and Haynes 230 [6]. It was also determined that the addition of V increases the hardness significantly (up to 11.4 GPa at 1150 °C) [7] and yield strength σ_0.2_ from 1058 MPa to 1246 MPa [5]. On the other hand, these alloys are brittle at room temperature, leading to significant limitations of their application as structural materials. Later, in Reference [8], it was shown that the addition of Ti to the NbMoTaW alloy might lead to an effective increase of the alloy plasticity up to 11.5% due to the increase of grain boundary cohesion. At present, there are known refractory elements-based HEAs with high wear-resistance [9], corrosion resistance [10], and plasticity [11].

Multi-component refractory elements-based alloys possess a mainly BCC structure. One of the most interesting HEA types is a system with coherent BCC and B2 phases with a microstructure similar to Ni-based alloys. The difference is that the disordered BCC phase is present as cubic nanoparticles. This structure provides a combination of high strength and high plasticity at high temperatures and is standard for Al-containing multi-component alloys. Thus, for the AlMo_0.5_NbTa_0.5_TiZr alloy, its yield strength is 745 MPa at 1000 °C, and good plasticity is preserved (the height decrease is >50% under compression) [12].

However, there can also be observed oxides and intermetallic phases, such as Laves phases, in the refractory HEAs that can affect the materials’ ductility. For example, in the NbCrMoTaTiZr, CrNbTiZr, and CrNbTiVZr alloys, the Laves phase is formed in the BCC-matrix because of the smaller radius of Cr in comparison to other elements [13,14,15]. Nevertheless, in Reference [2], an NbMoTaWVCr alloy with an extremely high yield strength of 3416 MPa and good plasticity of 5.3% at room temperature was obtained. Volume fractions of Laves phase (Cr,V)_2_(Ta,Nb) and oxide phase Ta_2_VO_6_ in this alloy were 7.7% and 6.2%, respectively. According to the authors, the high mechanical properties were achieved by forming an interstitial solid solution in the matrix and by fine grains (1.24 µm).

Modern powder metallurgy methods allow producing samples with submicron grains instead of a more common vacuum arc melting [16,17]. Ultrafine and nanosized powders of multi-component solid solutions can be synthesized by mechanical alloying (MA). Spark plasma sintering (SPS) allows the powders’ consolidation up to a high density in a few minutes. In Reference [18], the combined use of mechanical alloying and spark plasma sintering allowed obtaining samples of a high-entropy CoCrFeMnNi alloy with an average grain size of 270 nm.

The present work aimed to manufacture bulk samples of the refractory high-entropy alloy CrMoNbWV by MA and SPS and to investigate its properties. In this paper, the effect of sintering temperature on microstructure evolution, mechanical properties, wear and corrosion resistance of fabricated HEA is investigated.

## 2. Materials and Methods

Elemental powders (“NPK” Special Metallurgy”, Ekaterinburg, Russia) of Nb, Mo, W, V, and Cr (purity 99.9 %) were used to synthesize the high-entropy CrMoNbWV alloys. They were mixed in an equiatomic ratio. The particle size of the feedstock powders was 40–200 µm. Mechanical alloying was carried out in a planetary mill Fritsch Pulverisette 4 (FRITSCH GmbH, Idar-Oberstein, Germany), in an Ar atmosphere. The duration of MA was 5 h; the main disk/bowl rotation speed was 350/700 rpm; the milling ball material was high-strength steel; the milling ball diameter was 7–10 mm, and the average-weighted diameter was 8.6 mm; material to balls mass ratio was 1:20.

Sintering was carried out in a spark plasma sintering device HPD 25 (FCT Systeme GmbH, Rauenstein, Germany) in a graphite die of Ø40 mm at 1200, 1300, and 1400 °C. There was a pressure of 50 MPa and isothermal time at the maximal temperature of 5 min (samples further denoted at SPS 1200, 1300, and 1400).

X-ray diffraction analysis was carried out using a diffractometer Bruker D8 Advance (Bruker AXS Advanced X-ray Solutions GmbH, Karlsruhe, Germany) (CuKα = 0.15406 nm) in the 2Θ-range of 35–95 with a scanning step of 0.02 and exposition of 1.5 s at every step. Structural parameters were refined by the Rietveld method using the TOPAS5 program. Crystal density for equiatomic alloys was calculated from the mass and lattice parameter using the TOPAS5 program.

Particle morphology and structure were studied using a scanning electron microscope Mira 3 Tescan (Tescan Orsay holding, a.s., Brno-Kohoutovica, Czech Republic) while applying an acceleration voltage of 20 kV and working distance—20 mm. The powder particles’ chemical composition was determined on cross-sections by X-ray microanalysis using an Oxford INCA Wave 500 device (Oxford Instruments plc, Oxon, UK) in the scanning electron microscope.

Compressive strength characteristics were tested using a multifunctional tensile-testing machine Zwick/Roell Z100 (Zwick GmbH & Co. KG, Ulm, Germany), on cylindrical specimens (height—12 mm, diameter—6 mm). Measurements of the deformation were carried out on the traverse of the testing machine.

The microhardness was measured on a Buehler microhardness tester (Buehler Ltd., Lake County, IL, USA) with a 300 g load in the edge and 1000 g in the samples’ center. Measurements were carried out on the ground and polished specimens.

Wear tests were carried out, according to ASTM G65. As recorded by the standard, the testing device was equipped with a chlorobutyl rubber wheel (Peter the Great St. Petersburg Polytechnic University, St. Petersburg, Russia) with a hardness of A58-62. Silica sand with the following particle size distribution: 212–300 µm (not less than 95 %) and 300–425 µm (not more than 5%) was used as an abrasive material. Force against the sample was 130 N; the wheel revolutions number was 6000, and the lineal abrasion was 4309 m. A bulk sample loss referring to the path traveled by the testing bench rubber wheel was chosen as a comparative factor of the wear. The path traveled by the rubber wheel was calculated by accounting for the current perimeter of the rubber wheel and the required number of revolutions (6000 revolutions per test). The sample mass was measured before and after the tests with an accuracy of 0.0001 g. The wheel diameter was measured using a caliper with an accuracy of 0.05 mm.

Corrosion tests were carried out using the electrochemical method with a potentiostat VersaSTAT 4 (AMETEK, Inc., Berwyn, IL, USA), according to ASTM G3, G5, and G61. An Ag/AgCl_KCl sat_. electrode was used as a reference, and a platinum electrode was used as a counter electrode. The test procedure consisted of the following steps: the immersion of a flat sample with an area of about 1 cm^2^ into an experimental solution, the measurement of an open circuit potential (E_OCP_) for 55 min, and the provision of polarization with a scanning rate of 0.16 mV/s starting from E_OCP_-250 mV. The following type of solution was used: 0.5 M H_2_SO_4_. The tests were carried out at room temperature and ambient aeration. The following characteristics were determined: E_OCP_ and corrosion current density (i_corr_) obtained using the Tafel extrapolation method.

## 3. Results

**Figure 1:**
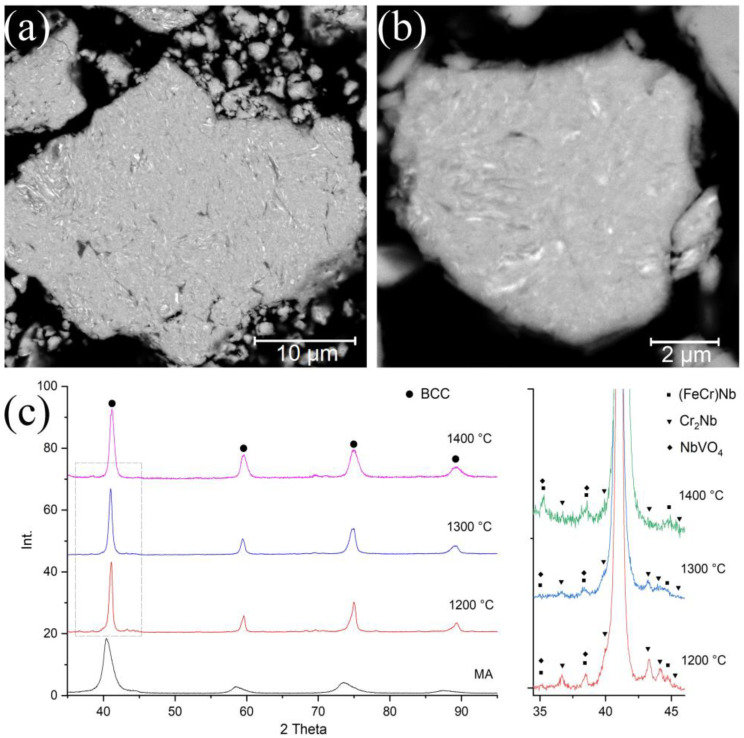
Microstructure (**a**,**b**) and phase composition (**c**) of high-entropy CrMoNbWV alloy powder after MA and SPS (at temperatures of 1200, 1300, 1400 °C).

Figure 1 shows the particle microstructure and the MA-powder phase composition. After 5 h of the MA, the synthesized powder possessed a homogeneous structure. At the BSE-SEM images, light layers with a thickness of 100–300 nm, enriched with W and Mo can be seen. Nevertheless, the distribution of the elements within the particle volume was homogeneous in general. The chemical composition of the MA-powder is presented in Table 1. The Fe content (3.5 wt.%) was caused by the milling yield of the milling balls’ material. The MA-powder possessed a BCC lattice with a parameter a = 3.146 Å. The feedstock elements belonged to the same group in the periodic system (V and VI), indicating the similarity of their electronic structures. Therefore, some of these components can fully dissolve in each other. The diffraction peak broadening was caused by a decrease in the size of coherent scattering regions and increased internal stresses due to intensive mechanical impact during the MA.

The bulk samples’ phase composition obtained by SPS at 1200–1400 °C is shown in Figure 1c. The XRD results show that the samples after sintering preserve a BCC structure, and its parameter slightly decreases (a = 3.106 Å) due to the separation of elements with small atomic radii (Cr, Fe, V) in the Laves phases and oxides. At the sintering temperature of 1200 °C, such Laves phases as Cr_2_Nb and (Fe, Cr)Nb, and such oxides mixture as NbVO_4_–VO precipitated. The increase in the sintering temperature led to a decrease in the Cr_2_Nb content. In the sample sintered at 1400 °C, this phase was almost absent.

**Figure 2:**
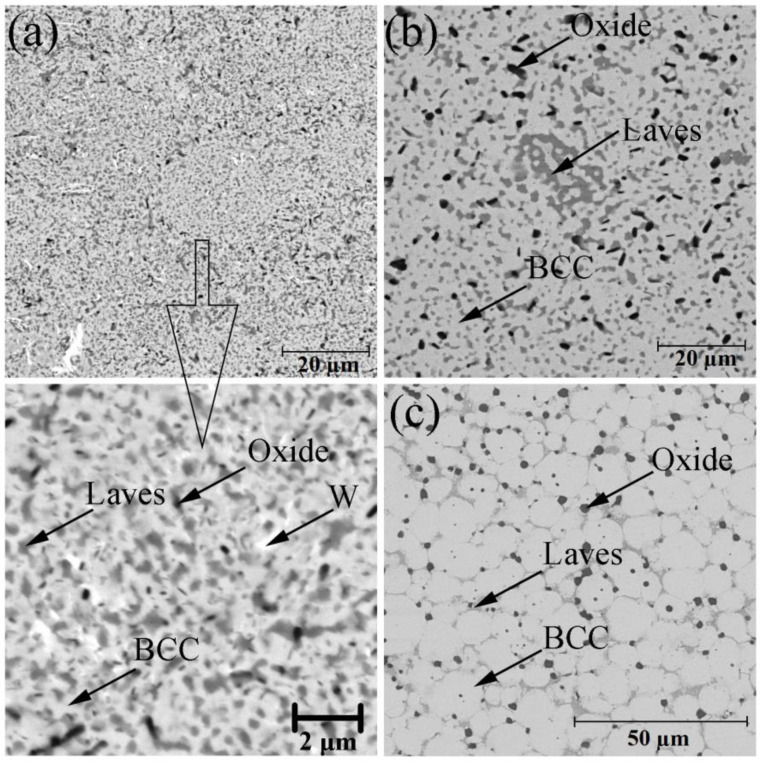
Microstructure of CrMoNbWV high-entropy alloy (HEA) samples, obtained by SPS at 1200 °C (**a**), 1300 °C (**b**), 1400 °C (**c**).

Figure 2 shows the BSE-images of the bulk samples’ microstructure. After sintering, there were three phase components, according to the difference in their contrasts. The light grey matrix was related to the BCC solid solution with the chemical composition close to the initial CrMoNbWV one. The grey regions along the grain boundaries were enriched with Nb, V, and O, and the chemical composition indicates the presence of the complex oxide NbVO_4_–VO. The contamination by oxygen might occur either during MA process or during discharging the powder. The chemical composition of the high-entropy CrMoNbWV alloy phases after SPS is represented in Table 2. It is shown that the HEA sample microstructure gradually changed with increased sintering temperature. The samples sintered at 1200 °C had a fine-crystalline structure without firm grain boundaries (Figure 2a). Therefore the analysis of the chemical composition of separate structural components was impossible to conduct. The temperature increase up to 1400 °C led to the samples’ recrystallization, resulting in equiaxial grains of a solid solution with a size of up to 15 µm (Figure 2c). In this case, the Laves phases partially dissolve in the matrix, and the oxides coagulate and localize along the recrystallized grain boundaries. It is worth noticing that the increase of the sintering temperature led to changes in oxide morphology: it became circular instead of splintery (polyangular).

The volume fraction of every phase was calculated by analyzing the SEM-images. In the sample sintered at 1200 °C, the Laves phases’ volume fractions and the oxides, distributed uniformly, were 41.6 and 4.7%, respectively. The increase of the sintering temperature led to a gradual decrease in the calculated volume fraction of the Laves phases, which was 21.5 and 11.5% at 1300 °C and 1400 °C, respectively. The calculated volume fraction of the oxides was constant and did not depend on the sintering temperature. The calculation results corresponded to the XRD results.

Calculation of the crystal density of equiatomic alloys by mass and lattice parameter showed that the CrMoNbWV HEA density with the lattice parameter a = 3.106 Å was 10.55 g/cm^3^. For the WNbMoTaV alloy, the crystal density at the lattice parameter a = 3.186 Å [19] was 12.42 g/cm^3^.

**Figure 3:**
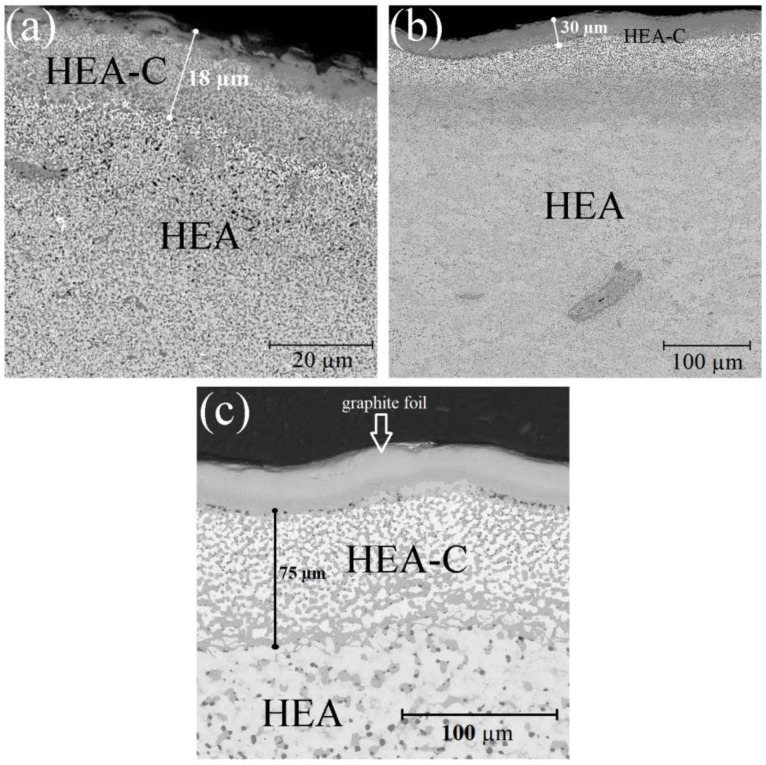
Carburized layer in CrMoNbWV HEA samples after SPS at 1200 °C (**a**), 1300 °C (**b**), 1400 °C (**c**).

When investigating the sample cross-sections in detail, it was determined that under SPS, the HEA powder interacted with the carbon of the equipment that led to carburization of the surface layer (Figure 3). The XRD showed that under sintering, carbon atoms were intercalated to the HEA BCC-lattice that led to strains in the latter and the formation of some HEA carbides. The carburized layer thickness depends on the sintering temperature and was 18–75 µm.

**Figure 4:**
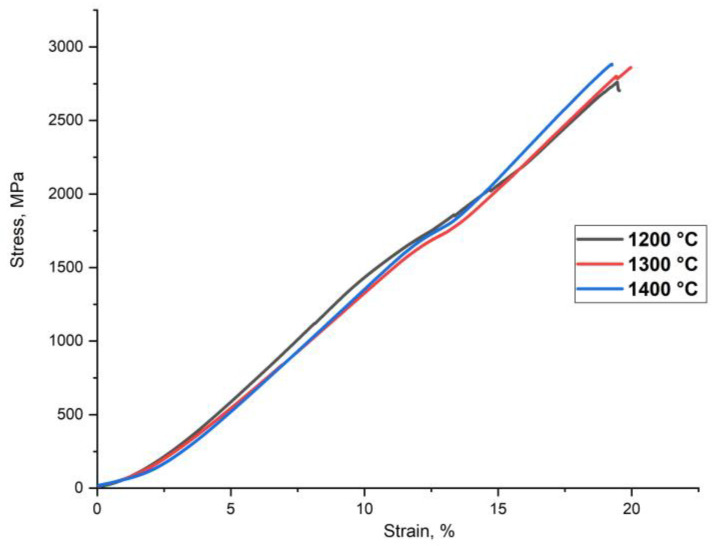
Compressive strength of the CrMoNbWV HEA samples obtained by SPS.

The SPS-samples possess specific gravity of 96–98%. Figure 4 represents the compressive deformation curves of the sintered samples at room temperature. The sintered samples exhibit the compressive strength of 2700–2870 MPa (Table 3). The samples’ low plasticity was caused by the high volume fraction of Cr_2_Nb, (Fe, Cr)Nb Laves phases, and NbVO_4_–VO oxides, which localized at the grain boundaries (Figure 2). These phases were brittle at room temperature, and their localization promoted a crack initiation and propagation along the grain boundaries resulting in a decrease of the HEA plasticity. The volume fraction of the Laves phases decreased in the sample sintered at 1400 °C. However, the oxide particle size significantly increases, as shown in Figure 2. The coarse inclusions can act as a crack initiation source and cause fractures in the BCC-matrix that leads to a decrease of the HEA plasticity. The increase of the sintering temperature does not significantly influence the sample strength. However, Figure 2 shows a growth of the BCC-matrix grains accompanies the increase in the sintering temperature, leading to a decrease of the hardness.

**Figure 5:**
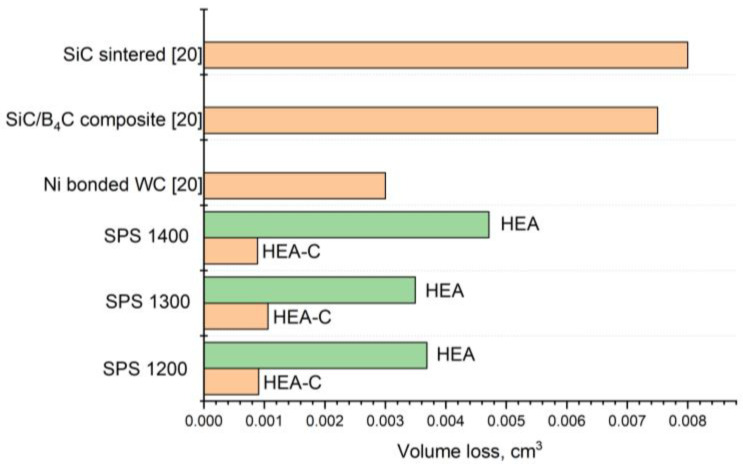
Bulk loss in the CrMoNbWV SPS-samples under the wear tests, according to ASTM G65.

Figure 5 represents the results of the bulk loss measurements under the wear tests, according to ASTM G65. The conducted research shows that the bulk loss of the sintered samples’ carburized layer was 0.001 cm^3^, which exceeded the values for nickel bonded tungsten carbide (0.003 cm^3^) [20]. After removing the carburized layer, the repeated tests were carried out, and the bulk loss was 0.0035–0.0048 cm^3^ in this case.

**Figure 6:**
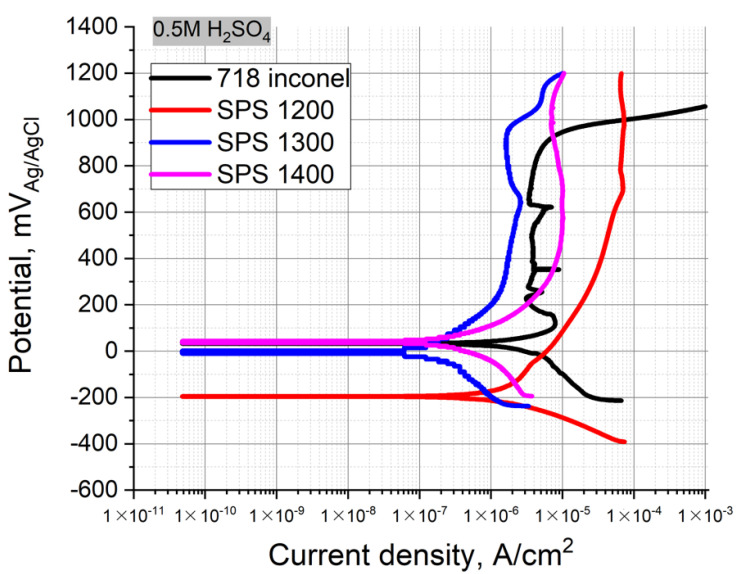
Polarization curves obtained for the CrMoNbWV HEA SPS-samples in 0.5 M H_2_SO_4_ solutions.

Figure 6 represents the polarization curves for the CrMoNbWV HEA SPS-samples in the 0.5 M H_2_SO_4_ used as electrolytes at room temperature. The values of E_OCP_ and i_corr_ are shown in Table 4. A corrosion and heat resistant Inconel 718 alloy was used as reference material.

All three samples exhibited good resistance to general corrosion in the sulfuric acid solution, i_corr_ reach 2.81×, 0.20, and 0.88 µA/cm^2^, respectively. The increase of the SPS temperature provided a positive shift of the E_OCP_ and a decrease of i_corr_, leading to the good corrosion resistance of the SPS samples obtained at 1300–1400 °C, in H_2_SO_4_ solution. 

**Figure 7:**
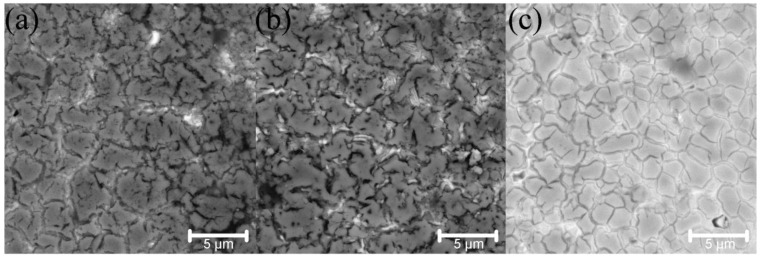
Corroded surface of CrMoNbWV HEA samples, obtained at 1200 °C (**a**), 1300 °C (**b**), 1400 °C (**c**).

Figure 7 represents the CrMoNbWV HEA SPS-samples surface after corrosion tests in the 0.5 M H_2_SO_4_ solution at room temperature. Surface after test showed a tendency to selective dissolution. It could be suggested that more probable locations for corrosion are (Fe, Cr)Nb and Cr_2_Nb Laves phases.

**Figure 8:**
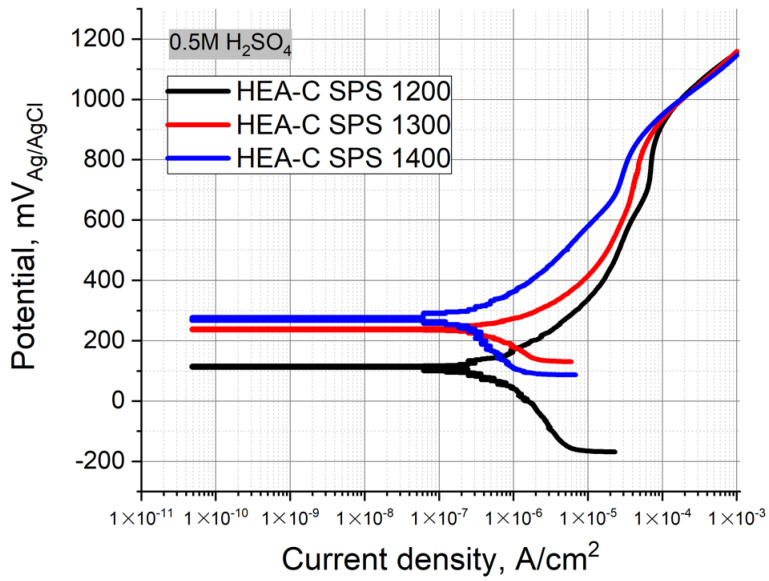
Polarization curves obtained for the carburized layer of the HEA SPS-samples in 0.5 M H_2_SO_4_ solution.

Figure 8 represents the Tafel polarization curves obtained for the carburized layer of the HEA SPS-samples. The electrolyte was the 0.5 M H_2_SO_4_ solution at room temperature. Table represents the values of E_OCP_ and i_corr._ The SPS temperature increase resulted in a decrease of i_corr_, where the values were 0.91, 0.66, and 0.21 µA/cm^2^. On the contrary, the E_OCP_ shifted to higher positive values from 100 to 276 mV, respectively. The polarization curves obtained for the carburized layers (denoted as HEA-C SPS 1200, 1300, and 1400) differed from each other and the bulk HEA curves. The carburized layers of the HEA SPS-samples obtained at 1200–1300 °C exhibited a narrow passivation region ΔE, and the potential increase up to >0.91 V resulted in a continuous increase of the current density that differed from the bulk HEA samples. The carburized layer of the HEA SPS-samples obtained at 1400 °C exhibited an almost linear increase of the current density under the increase of the potential. A clearly defined passivation plateau was almost absent, and the alloy components gradually dissolved, which results in general corrosion. The results showed that the chemical stability of the carburized layer increases with increasing sintering temperature.

## 4. Discussion

### 4.1. Phase Formation

Originally the HEA conception supposed the formation of a single-phase solid solution provided by high mixing entropy of the components. However, later studies on HEAs indicated that Δ*S_mix_* is not a single factor influencing the solid solution formation [21]. In addition, the common Hume–Rothery rule for binary systems is not to be applied for complex multi-component systems.

In Reference [22], parameter δ is proposed to estimate the stabilization of solid solutions. The parameter specifies the difference between atom radii of the elements, and the following rules are formulated to determine the solid solution formation: −20 ≤ Δ*H_mix_* ≤ 5 kJ/mol, 12 ≤ Δ*S_mix_* ≤ 17.5 J/K·mol, *δ* ≤ 6.4%.

(1) $$\Delta S_{mix}=-R\overset{n}{\underset{i=1}{\sum }}c_{i}\;ln\;c_{i}$$

(2) $$\Delta H_{mix}=\overset{n}{\underset{i=1,\;j\neq i}{\sum }}\Omega_{ij}c_{i}c_{j}$$

(3) $$\delta =\sqrt{\overset{n}{\underset{i=1}{\sum }}c_{i}{\left(1-\frac{r_{i}}{r_{av}}\right)}^{2}}$$

where *R*—absolute gas constant; $r_{i}$—i-component atomic radius; $c_{i}$—i-component concentration (at.%); $r_{av}$—average atomic radius; $\Omega_{ij}=4H_{mix}^{AB}$—parameter of interaction between i- and j-component in the solution.

According to the Miedema rule, Table 5 represents the mixing enthalpies of elemental pairs in the CrMoNbWV alloy calculated. Table 6 shows some physical and chemical properties of the elements.

Later in References [23,24], a critical value of the parameter *δ* was refined, and a more accurate thermodynamic parameter *Ω* was proposed. This parameter specifies the influence of mixing enthalpy and entropy and average melting point *T_m_* of the elements.

(4) $$\Omega =\frac{T_{m}\Delta S_{mix}}{\left|H_{mix}\right|}$$

(5) $$T_{m}=\overset{n}{\underset{i=1}{\sum }}c_{i}{\left(T_{m}\right)}_{i}$$

Reference [23] established that a single-phase solid solution was formed under the following conditions: *δ* ≤ 6.6% and *Ω* > 1.1. Table 7 represents the values of various parameters for the CrMoNbWV alloy.

The values in Table 7 find good agreement with the criteria mentioned above. Therefore a single-phase solid solution is expected to be formed in the CrMoNbWV alloy. To predict a solid solution structure, Reference [25] proposed using the average valence electron concentration (VEC).

(6) $$\mathrm{VEC}=\overset{n}{\underset{i=1}{\sum }}c_{i}{\left(\mathrm{VEC}\right)}_{i}$$

According to Reference [25], FCC-phases are stable at high VEC values (≥8). When the values are low (VEC ≤ 6.87), a BCC-structure is formed. According to the calculations (Table 7), if VEC for CrMoNbWV is 5.6, that suggests BCC solid solution formation.

In the present paper, the MA allowed to obtain a CrMoNbWV alloy with a single-phase BCC structure. However, the following SPS leads to partial decomposition of the solid solution and precipitation of the Laves phases (Cr_2_Nb, (Cr, Fe)Nb) and oxides (NbVO_4_-VO). It can be connected with significant Fe-impurities in the alloy. It is reported that the Laves phases are also formed in some other Cr-containing high-entropy alloys [2,14,15]. The smaller atomic radius of Cr can explain that in comparison to the other refractory elements. Niobium has the largest atomic radius among the alloying elements (Table 6), and chromium and iron have the smallest ones. The Laves phase precipitation reduces the atomic radii misfit and reduces the CrMoNbWV solid solution’s elastic energy. The Laves phase formation is consistent with the triple Nb-Cr-Fe phase diagram [26]. The precipitation of NbVO_4_ and VO is connected with the high affinity of these elements to oxygen. 

The increase of the SPS temperature leads to reverse dissolution of the Laves phases in the solid solution that is possibly connected with the higher influence of entropy component Δ*S_mix_* than the enthalpy one Δ*H_mix_* in this case. In addition, the increase of the sintering temperature promotes a higher diffusion of elements in the alloy.

### 4.2. Hardening and Wear

Prior research shows the mechanical properties of the refractory NbMoWVTa [5] and NbMoWVTaTi [27] HEAs obtained by arc melting, where the compressive strength of the alloys are 1270 and 2000 MPa, respectively. Refractory HEAs NbMoWVTa [19] and NbMoWVTaCr [2] obtained by MA and SPS showed higher compressive strengths of 3472 and 3834 MPa. It indicates that the compressive strength of the CrMoNbWV powder is significantly higher than that of the refractory HEAs obtained by melting methods but less than that obtained by MA and SPS. Simultaneously, the density of the CrMoNbWV alloy is lower than that of NbMoWVTa and NbMoWVTaCr HEAs. The high strength values of samples obtained by powder metallurgy methods can be explained by the influence of the following hardening mechanisms: (1) grain boundary hardening described by Hall–Petch relationship; (2) solid solution hardening due to strong lattice strains caused by substitutional elements; (3) solid solution hardening by interstitial elements; (4) Laves phases and oxides precipitations.

Compared to a coarse-grained structure of the HEA obtained by melting, the CrMoNbWV HEA, obtained by SPS of the MA-powder, has a structure with equiaxial grains with a size of less than 15 µm. This fact provides the grain boundary hardening of the BCC-matrix.

In this paper, the CrMoNbWV HEA samples were produced by powder metallurgy methods that inevitably lead to contamination by oxygen. Despite the precipitation of NbVO_4_–VO in the solid solution under the SPS, oxygen can still be partially dissolved in the BCC-matrix as an interstitial element. Interstitial elements, such as O, C, and N can strongly influence the solid solution’s hardening in refractory HEAs [28]. The Laves phases and oxides, formed under the SPS, are dispersed in the matrix and serve as barriers for dislocation motions. In addition, the precipitated oxides have a hexagonal lattice that is incoherent to the CrMoNbWV HEA BCC-matrix. It leads to the high strength of the material but simultaneously to the brittleness as well.

Simultaneously, the presence of the fine-grained Laves phases protects the surface from strong plastic deformations and prevents destructions during a sliding, while the BCC solid solution of high plasticity protects the surface from a brittle fracture. The increase of the SPS temperature results in a decrease of the Laves phase content in the samples. The Laves phase grains coarsen, leading to higher wear under the tests. The higher characteristics of the carburized layer probably result from the BCC-lattice strain caused by the intercalation of carbon atoms and the fine-dispersed HEA carbides. The difference in the wear rate for the carburized layer and the bulk HEA corresponds to the Archard wear equation. According to the equation, sliding wear resistance is proportional to the alloy hardness. The difference between the values for the carburized layer and the bulk HEA is 300%, indicating the superior wear resistance of the carburized layer compared to the conventional hard alloys. The bulk HEA exhibits wear resistance higher than a sintered SiC, or SiC/B4C composite does.

### 4.3. Corrosion Resistance

The conducted corrosion test shows that the CrMoNbWV HEA has good performance characteristics in the 0.5 M H_2_SO_4_ solution. The sintered samples exhibit a low corrosion rate connected with the strong passivation ability of Nb, Mo, V, and Cr. These elements can form protective passivating coatings on the alloy surface in acid solutions. Niobium is chemically resistant, and it does not dissolve in all conventional acids and their mixtures and exhibits a high tendency to passivation. Despite the relatively high negative standard potential of niobium, its inertness to acids can be explained by forming a thin dense protective film on its surface, and this film prevents a direct interaction between the metal and the solution. Anodic dissolution of niobium in aqueous electrolytes of various chemical compositions also fails due to the immediate passivation. Chromium is a principal alloying element in corrosion-resistant steels and Ni-based alloys. A chromium addition makes these alloys corrosion-resistant due to forming a protective passive film based on its oxide on the alloy surface [29]. Molybdenum improves the ability of passivation and resistance to pitting corrosion. It is considered that pure Mo forms a protective passive film based on MoO_2_ or can form MoO_4_^2−^ particles. However, in the HEA, Mo tends to form a σ-phase enriched with Mo and Cr, leading to their depletion in the matrix and negative influence on the HEA corrosion resistance. Like molybdenum, vanadium can form a passive film improving the corrosion resistance of the sample [29,30].

Nevertheless, some differences between the sintered samples in this paper are observed. The obtained SPS-samples have the same chemical composition, but there is a difference in phase composition and grain size depending on the sintering temperature. After the sintering, three main phase components are determined in all the samples. They are a BCC solid solution, Laves phases (Cr_2_Nb and (Fe, Cr)Nb), and an oxide mixture (NbVO_4_–VO). The increase of the sintering temperature results in a decrease of Cr_2_Nb content. In the sample sintered at 1400 °C, this phase is almost absent.

CrMoNbWV alloy shows the same trend of the opportunity of having increased corrosion resistance compared to traditional materials, in our case, Inconel 718. By comparing our alloy to commercial alloys, we can say that the values of corrosion currents densities obtained in 0.5 M H_2_SO_4_ are in the same range. The commercial alloys are slightly better. Corrosion tests of the VNbMoTaW alloy obtained using a pulsed direct current magnetron co-sputtering system showed that the corrosion current is in a 0.5 M H_2_SO_4_ solution 0.435 μA/cm^2^ [31], and the CrMoNbWV alloy (2.81 μA/cm^2^—SPS 1200, 0.20 µA/cm^2^—SPS 1300, 0.88 µA/cm^2^—SPS 1400).

As mentioned above, the HEA surface carburization under the SPS takes place. The increase in sintering temperature leads to increases of C due to a higher diffusion of C from the graphite crucible. Thus, the diffusion layer grows thicker, and the oxygen content decreases due to a more reductive atmosphere when the graphite crucible is used. The intercalation of carbon atoms to the HEA BCC structure leads to lattice strains and the formation of some amount of fine-dispersed HEA carbides. The formation of the fine-dispersed carbides in the samples probably provides the gradual dissolution of the alloy components and the almost total absence of the passivation region Δ*E*.

## 5. Conclusions

1. Refractory CrMoNbWV HEA was obtained from elemental powders by mechanical alloying followed by spark plasma sintering.
2. The phase composition, microstructure, and properties of the manufactured alloy depended strongly on the sintering conditions. It was determined that in the MA-powder with a BCC solid solution structure, the Laves phases Cr_2_Nb and (Fe, Cr)Nb precipitate under the SPS (41.6 vol.% at 1200 °C). Their volume fraction decreases when the SPS temperature increases and accounts for 21.5 vol.% at 1300 °C and 11.5 vol.% at 1400 °C. The presence of the oxides NbVO_4_–VO can be connected with the powder oxidation during the MA.
3. The sintered samples exhibit the compressive strength of 2700–2870 MPa. The samples’ low plasticity is caused by the high volume fraction of the Laves phases localized at the grain boundaries. In the sample sintered at 1400 °C, the volume fraction of the Laves phases is lowered. However, the particle size of the oxides increases significantly. The coarse inclusions can serve as sources of crack initiation in the BCC-matrix.
4. The synthesized CrMoNbWV HEA exhibited high corrosion resistance, hardness, and wear resistance. A promising way to use the CrMoNbWV HEA is to apply it as friction couples and for hard abrasive wear conditions in corrosive environments.
5. The CrMoNbWV HEA has comparable mechanical and corrosive properties with the WNbMoTaV type HEA, but at the same time has a reduced density: CrMoNbWV—10.55 g/cm^3^, WNbMoTaV—12.42 g/cm^3^.

This research demonstrated that refractory CrMoNbWV HEA could be successfully fabricated by mechanical alloying and spark plasma sintering. Further research will be aimed at obtaining a powder without iron, studying the duration effect of the SPS process, and additional post-process heat treatment on the alloy’s structure and properties.

## Figures and Tables

**Table 1:** Chemical composition of the MA-powder, at.%.

Sample	Nb	Mo	W	V	Cr	Fe	O
MA	18.22	19.32	17.63	17.11	17.57	5.54	4.62

**Table 2:** Chemical compositions of the phases in CrMoNbWV HEA after SPS, at.%.

Sample	Phase	Volume Fraction, %	Nb	Mo	W	V	Cr	Fe	O
SPS 1200	BCC	53.6	18.27	19.36	17.45	17.08	17.49	5.47	4.88
	Oxide	4.8							
	Laves phase	41.6							
SPS 1300	BCC	74.7	17.64	22.78	21.13	17.63	17.79	3.03	-
	Oxide	3.8	7.88	-	-	31.64	-	-	60.48
	Laves phase	21.5	28.96	-	-	-	37.92	33.12	-
SPS 1400	BCC	83.6	18.02	21.81	20.93	16.36	17.19	5.69	-
	Oxide	4.9	9.47	-	-	33.13	-	-	57.40
	Laves phase	11.5	45.26	-	-	-	30.61	24.13	-

**Table 3:** Mechanical characteristics at room temperature of CrMoNbWV HEA after SPS.

Sample	Compressive Strength, MPa	Microhardness, HV	
		Edge	Center
SPS 1200	2700	1072 ± 22	971 ± 14
SPS 1300	2860	1154 ± 17	1009 ± 12
SPS 1400	2875	1266 ± 66	746 ± 19

**Table 4:** Corrosion test results for CrMoNbWV HEA after SPS.

Sample	E_OCP_, mV	i_corr,_ µA/cm^2^
Inconel 718	43	3.88
SPS 1200	−178	2.81
SPS 1300	0	0.20
SPS 1400	34	0.88
HEA-C SPS 1200	100	0.91
HEA-C SPS 1300	228	0.66
HEA-C SPS 1400	276	0.21

**Table 5:** Equiatomic pair mixing enthalpies $H_{mix}^{AB}$ (kJ·mol^−1^).

Element	Nb	Cr	W	V	Mo
Nb	-	−7.12	−8.52	−1.03	−5.58
Cr	-	-	0.95	−1.94	0.38
W	-	-	-	−0.793	−0.22
V	-	-	-	-	0.01
Mo	-	-	-	-	-

**Table 6:** Properties of alloying elements.

Element	Atomic Radius r, Å	Melting Point *T_m_*, K	VEC
Nb	1.43	2741	5
Cr	1.25	2130	6
W	1.37	3695	6
V	1.31	2160	5
Mo	1.36	2902	6

**Table 7:** Parameters of CrMoNbWV alloy.

Composition	Δ*H_mix_*, kJ/mol	Δ*S_mix_*, J/K·mol	δ, %	*Ω*	VEC
CrNbMoVW	−3.82	13.37	4.5	9.65	5.6

## Data Availability

The data presented in this study are available on request from corresponding author.
